# High Prevalence of CTX-M Type Extended-Spectrum Beta-Lactamase Genes and Detection of NDM-1 Carbapenemase Gene in Extraintestinal Pathogenic *Escherichia coli* in Cuba

**DOI:** 10.3390/pathogens9010065

**Published:** 2020-01-16

**Authors:** Dianelys Quiñones, Meiji Soe Aung, Yenisel Carmona, María Karla González, Niurka Pereda, Mercedes Hidalgo, Mayrelis Rivero, Arnaldo Zayas, Rosa del Campo, Noriko Urushibara, Nobumichi Kobayashi

**Affiliations:** 1 Department of Bacteriology-Mycology, Pedro Kourí Institute of Tropical Medicine, Havana 17100, Cuba; dianymariam@gmail.com (D.Q.); yeniselc@ipk.sld.cu (Y.C.); marikarla@ipk.sld.cu (M.K.G.); niurkap@ipk.sld.cu (N.P.); 2 Department of Hygiene, Sapporo Medical University School of Medicine, Sapporo 060-8556, Japan; meijisoeaung@sapmed.ac.jp (M.S.A.); noriko-u@sapmed.ac.jp (N.U.); 3 Microbiology Department, Héroes de Baire Hospital, Isla de La Juventud 25100, Cuba; merci8009@nauta.cu; 4 Microbiology Department, Enrique Cabrera hospital, Havana 10400, Cuba; dia@ipk.sld.cu; 5 Microbiology Department, Juan Bruno Zayas Hospital, Santiago de Cuba 90100, Cuba; arnaldo.zayas@infomed.sld.cu; 6 Ramón y Cajal Hospital, 28002 Madrid, Spain; rosacampo@yahoo.com

**Keywords:** extraintestinal pathogenic *E. coli* (ExPEC), extended-spectrum beta-lactamase (ESBL), carbapenemase, NDM-1, Cuba

## Abstract

Increase of extraintestinal pathogenic *Escherichia coli* (ExPEC) showing resistance to beta-lactams is a major public health concern. This study was conducted as a first molecular epidemiological study on ExPEC in Cuba, regarding prevalence of extended-spectrum beta-lactamases (ESBLs) and carbapenemase genes. A total of 306 ExPEC isolates collected in medical institutions in 16 regions in Cuba (2014–2018) were analyzed for their genotypes and presence of genes encoding ESBL, carbapenemase, plasmid-mediated quinolone resistance (PMQR) determinants by PCR and sequencing. The most common phylogenetic group of ExPEC was B2 (49%), followed by D (23%), A (21%), and B1 (7%). Among ESBL genes detected, *bla*_CTX-M_ was the most common and detected in 61% of ExPEC, with *bla*_CTX-M-15_ being dominant and distributed to all the phylogenetic groups. NDM-1 type carbapenemase gene was identified in two isolates of phylogenetic group B1-ST448. Phylogenetic group B2 ExPEC belonged to mostly ST131 (or its single-locus variant) with O25b allele, harboring *bla*_CTX-M-27_, and included an isolate of emerging type ST1193. *aac (6’)-Ib-cr* was the most prevalent PMQR gene (40.5%), being present in 54.5% of CTX-M-positive isolates. These results indicated high prevalence of CTX-M genes and the emergence of NDM-1 gene among recent ExPEC in Cuba, depicting an alarming situation.

## 1. Introduction

*Escherichia coli* is the most representative Gram-negative bacteria in the intestinal tracts of humans and animals as a commensal organism. However, infectious diseases in humans are caused by a group of *E. coli* strains, e.g., pathogenic *E. coli*, which is classified into diarrheagenic *E. coli* and extraintestinal pathogenic *E. coli* (ExPEC). While both pathogenic *E. coli* are distributed globally, ExPEC has been described as the common cause of community-acquired urinary tract infections and bloodstream infections [1]. Among the four major phylogenetic groups (i.e., A, B1, B2, D), ExPEC strains mainly belong to group B2, and to a lesser extent, group D. Although ExPEC consists of strains with many lineages, only a subset of lineages represented by sequence type (ST) 131 (ST131) is responsible for majority of infections in humans and considered a pandemic multiresistant clone [2]. 

Acquisition of drug resistance by ExPEC, as well as spread of multidrug-resistant ExPEC, has been established as a global public health concern. Most importantly, increased prevalence of the *E. coli* resistant to beta-lactam antibiotics raises morbidity and mortality with social costs [3,4]. Beta-lactamases that represent the most common cause of resistance to this class of antimicrobials have been classified into four major groups; penicillinases, cephalosporinases, extended-spectrum beta-lactamases (ESBLs), and carbapenemases. ESBLs hydrolyze all the beta-lactams except carbapenems and cephamycins, and are inactivated by beta-lactamase inhibitors such as clavulanic acid, sulbactam or tazobactam. ESBLs consist of three main types, TEM, SHV, and CTX-M, among which CTX-M is dominant and has become disseminated globally, with CTX-M-14 and CTX-M-15 being the major genotypes [5]. In addition to ESBL, AmpC beta-lactamases are also responsible for resistance to broad-spectrum cephalosporins [6]. Particularly, plasmid-mediated AmpC (pAmpC) carried by *E. coli* and other *Enterobacteriaceae* species has become a major clinical concern due to its resistance traits and transferability [7]. Carbapenem resistance in *Enterobacteriaceae* is primarily mediated by carbapenemases, which have been classified into Ambler class A (KPC, IMI, GES types), B (NDM, IMP, VIM types), and D (OXA types) enzymes [8]. Among them, class B carbapenemases (metallo beta-lactamases) exhibit a broad spectrum of activity to all penicillins, cephalosporins and carbapenems except for aztreonam. NDM is recognized as an emerging class B carbapenemase, and NDM-producing *E. coli* have been reported around the world [9]. 

Fluoroquinolone-resistant *E. coli* has been increasing as a uropathogen globally, following the extensive use of this antibiotics, often associated with the increasing trend of ESBL-producing ExPEC [10]. Resistance to fluoroquinolone is caused primarily by occurrence of mutation(s) in GyrA subunit of DNA gyrase and ParC subunit of topoisomerase IV. In addition, plasmid-mediated quinolone resistance (PMQR) determinants represented by Qnr, aac (6’)-Ib-cr, and QepA confer reduced susceptibility to fluoroquinolones [11].

In Cuba, drug resistance and prevalence of beta-lactamase genes in ExPEC has been scarcely studied to date, and mechanism of fluoroquinolone resistance has never been analyzed. Although only an available report showed prevalence of TEM and SHV for hospital isolates collected from 2002 to 2004 [12], there has been no report in Cuba regarding prevalence and types of beta-lactamase genes and genotypes of *E. coli* showing resistance to beta-lactams. The present study was conducted to investigate drug resistance, prevalence and genetic characteristics of recent ExPEC isolates harboring ESBL, pAmpC, carbapenemase genes and PMQR genes from whole regions in Cuba. We report here high prevalence of ST131 ExPEC carrying CTX-M-15 or CTX-M-27 genes, and first identification of NDM-1 gene in ST448 *E. coli* in Cuba.

## 2. Results

Among a total of 306 *E. coli* isolates, the dominant phylogenetic group was B2 (49%), followed by group D (23%) and A (21%) (Table 1). Resistance rates to 18 antimicrobials in the decreasing order are as follows: nalidixic acid (77.4%), norfloxacin (77.1%), cefotaxime (76.1%), ciprofloxacin (76.1%), cefuroxime (73.9%), ceftazidime (70%), cefepime (68.3%), trimethoprim-sulfamethoxazole (67%), aztreonam (53%), gentamicin (44.7%), tobramycin (40.7%), cefoxitin (22%), piperacillin-tazobactam (17%), amikacin (6.9%), fosfomycin (5.5%), meropenem (5.2%), imipenem (2.3%) and colistin (0.7%). 

The most prevalent beta-lactamase gene was *bla*_CTX-M_ (61.1%), followed by *bla*_TEM_ (31.7%) while the pAmpC gene (*bla*_CMY-2_) and carbapenemase gene (*bla*_NDM-1_) were detected in four (1.4%) and two (0.7%) isolates, respectively (Table 1). The two NDM-1-positive isolates were derived from patients in different provinces, although information of their overseas travel history was not available. Most CTX-M gene belonged to CTX-M-1 group which was found in 54.9% of all isolates and accounted for 90% of *bla*_CTX-M_ genes. CTX-M-1 group beta-lactamase gene was detected in all the phylogenetic groups at the rate of 52–59% (phylogenetic group A, 57.8%; B1, 52.4%; B2, 52%; D, 59%). Most of *bla*_CTX-M_-_9_ group was found in phylogenetic group B2 isolates, while *bla*_CTX-M-2_ group was detected in two isolates of group A. TEM gene was distributed to all the phylogenetic groups. More than half of TEM gene (54.6%, 53/97) was associated with CTX-M gene. Accordingly, 17.3% of ExPEC isolates had both *bla*_TEM_ and *bla*_CTX-M_. CMY-2 and NDM-1 genes were identified in four and two isolates, respectively. Carbapenemase genes encoding VIM, IMP, KPC, and OXA-48 were not detected.

Five PMQR genes (*aac (6)’-Ib-cr*, *qnrB*, *qnrD*, *qnrS*, and *oqxAB*) were detected, with *aac (6’)-Ib-cr* being the most common (40.5% of all the isolates), followed by *qnrB* and *qnrS* (Table 1). Detection rate of *aac (6’)-Ib-cr* was significantly higher among phylogenetic group B2 (47%) (*p* < 0.05), than group A (42%), B1 (38%), and D (25%). O25b allele was detected in only phylogenetic group B2 with a rate of 70.7% (106/150). Isolates positive for both *aac (6’)-Ib-cr* and *bla*_CTX-M_ accounted for 33% (n = 102) of all isolates. Among CTX-M-positive isolates, prevalence of *aac (6’)-Ib-cr* was 54.5%, which was higher than those of *qnrB* and *qnrS* (Table S1). 

Genotypes of *E. coli* (ST, *fimH*) and beta-lactamases were determined for a total of 71 ExPEC isolates consisting of 16, 11, 30, and 14 isolates of phylogenetic groups A, B1, B2, and D, respectively, and summarized in Table 2. These isolates were selected from different provinces and different specimens, in each year, and included ExPEC harboring one of the six CTX-M genes (group 1, *bla*_CTX-M-15_, *bla*_CTX-M-32_, *bla*_CTX-M-55_; group 2, *bla*_CTX-M-2_; group 9, *bla*_CTX-M-14,_ *bla*_CTX-M-27_), and those with TEM (*bla*_TEM-1_), CMY (*bla*_CMY-2_), or NDM (*bla*_NDM-1_). Common STs of phylogenetic group A isolates were ST10, ST410, and their single-locus variant (SLV) and double-locus variant (DLV), and these isolates harbored mostly *bla*_CTX-M-15_ and any of the PMQR genes. ST448 was detected in only phylogenetic group B1 ExPEC, and two ST448 isolates possessed NDM-1 gene. Isolates with ST405 and its variants were commonly identified among phylogenetic group D. Except for only a single isolate of ST1193, all the phylogenetic group B2 isolates belonged to ST131 or its SLVs (five STs), and harbored *bla*_CTX-M-15_ or *bla*_CTX-M-27_. Eighteen isolates were positive for O25b allele, and classified into *fimH*-30 type. Among the five ST131 SLV detected in phylogroup B2, three STs (ST5716, ST5717, ST5718) were newly identified in the present study.

Mutations in QRDR in GyrA and ParC were analyzed for 39 isolates showing resistance to quinolones (Table S2). In most isolates, mutations were detected in both proteins. S83L, D87N mutations in GyrA and S80I, E84V mutations in ParC were the most commonly identified. Among the 22 isolates with double mutations in both GyrA and ParC, 14 isolates harbored *aac (6’)-Ib-cr*. Two isolates belonging to phylogenetic group B2 showed resistance to colistin (MIC, 16 and 8 mg/μL), while *mcr-1*, *mcr-2*, *mcr-3* genes were not detected by PCR.

## 3. Discussion

In the present study, we observed high prevalence of *E. coli* positive for CTX-M gene (61%) as well as those resistant to ceftazidime (70%) and cefotaxime (76.1%). In a surveillance report from 11 Latin American countries (2011–2014), documented rates of CLSI ESBL screening phenotype in *E. coli* ranged from 14.7% (Brazil) to 69.9% (Mexico), while overall rate was 37.7% [13]. These rates were higher than previous surveillance (2008–2010) in the four Latin American countries representing ESBL rates as 18.1–48.4% [14]. Comparing these CTX-M-positive rates with those in our present study (2014–2018), Cuba is considered one of the countries showing highest prevalence of ESBL in *E. coli* among Latin America. The early study in Cuba (2002–2004, Havana city) reported that ESBL phenotype rate was 10%, associated with resistance rates to cefotaxime as 14.1% [12]. Accordingly, ESBL-producing *E. coli* is suggested to have increased drastically during the past decade in Cuba, similarly to the increasing trend in other Latin American countries. 

In Cuba, CTX-M type beta-lactamase is considered to be virtually a predominant ESBL, because all the TEM genes analyzed were assigned to non-ESBL genotype (TEM-1). Among the CTX-M type beta-lactamase genes, *bla*_CTX-M-15_ was the dominant type, as reported in the United States [15] and Canada [16], and distributed to all the phylogenetic groups. Phylogenetic group B2 included ST131 *E. coli* with O25b allele harboring *bla*_CTX-M-15_, which is known as the pandemic clonal group of multidrug resistant ExPEC [17], was revealed to be prevalent in Cuba in our present study. Furthermore, it was notable that *bla*_CTX-M-27_ was detected in B2-ST131 isolates showing higher incidence than *bla*_CTX-M-15_. ST131 *E. coli* with *bla*_CTX-M-27_ has been rapidly increasing in the past decade in Asia, Europe, and north America [18]. We found unusually high prevalence of ST131 ExPEC with *bla*_CTX-M-27_, suggesting the need for further monitoring of this clone. In addition, rare CTX-M-types, *bla*_CTX-M-32_ and *bla*_CTX-M-55_ were detected in phylogenetic group A isolates. CTX-M-55 has been reported as an emerging ESBL type among humans, animals, and the environment. [19], while *bla*_CTX-M-32_ is often associated with cattle and meat products [20]. Recently in Cuba, *bla*_CTX-M-32_ was identified in plasmid of an *E. coli* strain isolated from a healthy pig [21]. Hence, there may be a possibility that *E. coli* having CTX-M-32 and -55 genes might be derived from animal or environmental origin. 

Since the first identification of NDM-1 in 2008, NDM-type carbapenemase has attracted worldwide attention because of its rapid dissemination among Gram-negative bacteria that caused infections or colonization in human and animals, and also those distributed to environments [22]. Main reservoir of NDM producers is regarded as South Asia, while secondary reservoir is considered the Balkan regions and the Middle East [23]. Among NDM variants that have been discriminated into more than 20 types, NDM-1 is the most common globally, and distributed mainly to specific clones (53 STs) of *E. coli* with ST101, ST167, ST131, ST405, ST40, and ST648 being more frequently identified. In Latin America, prevalence of carbapenem-resistant *E. coli* has been extremely low [13,24], and *E. coli* carrying NDM gene has been rarely reported [25]. Only isolates of ST10 and ST617 *E. coli* from nosocomial outbreaks were reported to harbor *bla*_NDM-1_ in Mexico [25,26]. In our present study, NDM-1 gene was identified in two ExPEC isolates of phylogenetic group B1-ST448 that were isolated from sporadic infections in 2016, representing NDM-detection rate of 0.7% (2/306). This is the first report of NDM-1 gene in *E. coli* in Cuba, although we identified *bla*_NDM-1_ in a rare *Acinetobacter* species, *A. soli* in 2011 [27]. It was also remarkable in the present study that *bla*_NDM-1_ was detected in a rare *E. coli* clone, ST448. This clone harboring NDM gene has been identified in only limited reports in Asia (The Middle East and India) and Europe (Spain, Poland), and associated with various NDM-types, with NDM-5 being common [22,28,29]. Furthermore, KPC-3 and VIM-1-producing ST448 *E. coli* was reported in Spain, as a unique multiresistant clone [30]. Although it is not certain whether the Cuban NDM-producing ST448 *E. coli* was derived from Europe or autochthonous infection, it is necessary to carefully monitor the trend of this novel clone. 

While phylogenetic group B2 isolates were mostly assigned into ST131 or its variant, it was worthy of note that a single isolate (IPK-629) belonged to ST1193, which is described as an emerging clone [31]. ST1193 belongs to the ST14 clonal complex, and shows fluoroquinolone resistance, while resistance profiles were variable depending on isolates [31]. This *E. coli* clone was documented to show temporal prevalence trend in the US [32], and ST1193 having CTX-M-14 and CTX-M-15 genes were reported in Germany [33], and only the latest report from France described CTX-M-27 gene-positive ST1193 *E. coli* [34]. IPK-629 in our present study was isolated from lochia in 2018, and had CTX-M-27 gene, and showed resistance to ceftazidime, ciprofloxacin, and trimethoprim-sulfamethoxazole. ST1193 *E. coli* was suggested to have evolved through frequent gain or loss of resistance gene cassettes [31], and thus, possible to change in its resistance profiles and epidemiological features in the future. Therefore, detection of ST1193 in Cuba may be a concern for the control of ExPEC. 

In the present study, high resistance rate was noted also against ciprofloxacin (76.1%), associated with mutations in GyrA and ParC and prevalence of PMQR gene *aac (6‘)-Ib-cr*. This finding suggests substantial progress in resistance of *E. coli* to fluoroquinolone in Cuba. It has been described that PMQR gene *aac (6‘)-Ib-cr* is often located on plasmids of the IncF family with *bla*_CTX-M-15_ [11]. Considerable rate of *aac (6‘)-Ib-cr* among CTX-M-positive *E. coli* (54.5%) observed in our present study suggest coexistence of these genes on plasmids, which may imply the spread of ESBL associated with quinolone and aminoglycoside resistance, leading to dissemination of multidrug resistant *E. coli*. While *qnrB* has been implicated in ISCR-1-linked genes encoding CTX-M and carbapenemases on plasmids [35], prevalence of *qnrB* was low among CTX-M-positive isolates in our study. Furthermore, among nine *qnrS*-positive isolates, only two isolates had TEM genes, although association of *qnrS1* with *bla*_TEM_ was documented [35]. These findings suggest the presence of an uncommon or novel type of plasmid containing *qnr* genes among ExPEC.

Our present study revealed high prevalence of CTX-M type ESBL gene represented by *bla*_CTX-M-15_ in ExPEC in Cuba, associated with progress of quinolone resistance, and described also first identification in *E. coli* of NDM-1 type carbapenemase gene. These findings could be attributable to the presumptive frequent use of third generation cephalosporins and carbapenems in Cuba. Along with continuous surveillance of antimicrobial resistance, a systematic investigation for the usage, i.e., frequency and number of individual antimicrobials used in this country would be necessary for the effective control of antimicrobial resistance in ExPEC.

## 4. Materials and Methods 

### 4.1. Bacterial Isolates

A total of 306 non-duplicate clinical isolates of *E. coli* derived from patients with extraintestinal infections were analyzed. These isolates were collected from hospitals in 16 regions (provinces) in Cuba during a period between 2014 and 2018. The main source of the isolates was urine (49.7%), followed by blood (13.4%), wound (10.8%), tracheal aspirate (9.5%), skin (5.6%), cerebrospinal fluid (2.9%), catheter tip (2.3%), and others (5.8%). Bacterial identification was performed by conventional method. Gram-negative rods grown on MacConkey agar were identified by colonial morphology, motility and a series of biochemical tests (indole test, citrate utilization, urease test, oxidase reaction, lysine and ornithine test and sugar fermentation). Further, *E. coli* was confirmed by PCR targeting *adk* using primers employed in multilocus sequence typing (MLST) of this bacterial species [36]. 

### 4.2. Susceptibility Testing

Minimum inhibitory concentration (MIC) was measured for 18 antimicrobial agents (amikacin, aztreonam, ceftazidime, ciprofloxacin, colistin, cefotaxime, cefuroxime, cefepime, fosfomycin, cefoxitin, gentamicin, imipenem, meropenem, nalidixic acid, norfloxacin, sulfamethoxazole/trimethoprim, tobramycin, piperacillin-tazobactam). E-test was employed for beta-lactams, while disc diffusion test for other antibiotics except for colistin. Broth microdilution test was used for colistin, and also for imipenem and meropenem for confirmation of MIC. Susceptibility to all the antimicrobials except for nalidixic acid was judged according to EUCAST guideline [37]. CLSI guideline [38] was employed for nalidixic acid, of which breakpoint was not assigned in EUCAST guideline.

### 4.3. Molecular Detection of Beta-Lactamase Genes and Plasmid-Mediated Quinolone Resistance (PMQR) Genes 

For all the *E. coli* isolates, presence of beta-lactamase genes *bla*_CTX-M_, *bla*_TEM_, *bla*_SHV_ was examined by multiplex PCR as described previously [39], and four *bla*_CTX-M_ subgroups (group 1, 2, 9 and 8/25/26) were discriminated by multiplex PCR assay [40]. For all the isolates showing resistance to imipenem and/or meropenem, presence of carbapenemase genes (*bla*_NDM_, *bla*_VIM_, *bla*_IMP_, *bla*_KPC_, and *bla*_OXA-48_) were confirmed by multiplex/uniplex PCR using primers and conditions as described previously [41]. Plasmid-mediated AmpC beta-lactamase genes consisting of six families were detected by multiplex PCR according to the scheme described by Perez-Perez and Hanson [42]. Nucleotide sequences of full-length *bla*_TEM_, *bla*_CTX-M_, carbapenemase genes (*bla*_NDM_), and AmpC genes (*bla*_CMY_) were determined directly from PCR products with primers listed in Table S3, using the BigDye Terminator v3.1 Cycle Sequencing kit (Applied Biosystems, Foster City, CA, USA) on an automated DNA sequencer (ABI PRISM 3100). Subtypes of beta-lactamase genes were determined by using standard nucleotide BLAST (Basic Local Alignment Search Tool) available at the NCBI website [43]. Identification of plasmid-mediated quinolone resistance (PMQR) genes (*aac (6’)-Ib-cr*, *qnrA*, *qnrB*, *qnrC*, *qnrD*, *qnrS*, *oqxAB* and *qepA*) was also performed by multiplex PCR using primers and conditions as described previously [44].

### 4.4. Genetic Analysis of E. coli

Four main phylogenetic groups of *E. coli* (A, B1, B2, and D) were discriminated by triplex PCR method described by Clermont et al. [45]. Sequence type (ST) of *E. coli* based on Achtman scheme of MLST was assigned by determination of partial sequence of seven housekeeping genes (*adk*, *fumC*, *gyrB*, *icd*, *mdh*, *purA* and *recA*) [36]. Presence of O25b allele was confirmed by PCR as described previously [46], and isolates with O25b allele were further analyzed for genotype based on *fimH* (type 1 fimbrial adhesin gene) by PCR and direct sequencing [47], using the FimTyper 1.0 web-based tool. Presence of mutation in quinolone-resistance determining region (QRDR) of DNA gyrase (GyrA) and topoisomerase IV (ParC) was analyzed for selected quinolone-resistant isolates by PCR and direct sequencing. Primer sequences for PCR are as follows: *gyrA*, forward 5′-ACGTACTAGGCAATGACTGG-3′, reverse 5′-AGTCGCCGTCGATAGAA-3′; *parC*, forward 5′-TGTATGCGATGTCTGAACTG-3′, reverse 5′-CTCAATAGCAGCTCGGAATA-3′. For isolates showing resistance to colistin, detection of *mcr* genes was attempted by PCR, as described previously [24]. 

### 4.5. GenBank Accession Numbers

The nucleotide sequence of beta-lactamase genes encoding TEM-1, CTX-M-2, -14, -15, -27, -32, and -55, and CMY-2 were deposited in the GenBank database under accession numbers MH900520-MH900529.

## Figures and Tables

**Table 1:** Prevalence of beta-lactamase genes and plasmid-mediated quinolone resistance (PMQR) genes in *E. coli* isolates (n = 306, 2014–2018).

Beta-Lactamase Gene (Genotype) / PMQR Determinant * ^1^ / O25b Allele		Number of Isolates in Phylogenetic Group (%)				Total
		A (n = 64)	B1 (n = 21)	B2 (n = 150)	D (n = 71)	(n = 306) (%)
CTX-M		39 (60.9)	11 (52.4)	94 (62.7)	43 (60.6)	187 (61.1)
	CTX-M-1 group	37 (57.8)	11 (52.4)	78 (52)	42 (59.2)	168 (54.9)
	CTX-M-2 group	2 (3.1)	0	0	0	2 (0.7)
	CTX-M-9 group	0	0	16 (10.7)	1 (1.4)	17 * ^2^ (5.6)
TEM		16 (25)	7 (33.3)	47 (31.3)	27 (38.0)	97 (31.7)
	CTX-M * ^3^ + TEM	8 (12.5)	5 (23.8)	26 (17.3)	14 (19.7)	53 (17.3)
NDM-1		0	2 (9.5)	0	0	2 (0.7)
CMY-2		1 (1.6)	3 (14.3)	0	0	4 (1.3)
PMQR						
	*aac (6’)-Ib-cr*	27 (42.2)	8 (38.1)	71 (47.3)	18 (25.4)	124 (40.5)
	*qnrB*	16 (25)	3 (14.3)	5 (3.3)	17 (23.9)	41 (13.4)
	*qnrD*	1 (1.6)	0	1 (0.7)	0	2 (0.7)
	*qnrS*	4 (6.3)	1 (4.8)	2 (1.3)	2 (2.8)	9 (2.9)
	*oqxAB*	2 (3.1)	0	0	0	2 (0.7)
	CTX-M * ^4^ + *aac (6’)-Ib-cr*	22 (34.4)	7 (33.3)	57 (38)	16 (22.5)	102 (33.3)
O25b allele		0	0	106 (70.7)	0	106 (34.6)

* ^1^ Following genes were not detected in any isolate: *qnrA*, *qnrC* and *qepA*. * ^2^ CTX-M-27, 16 isolates; CTX-M-14, 1 isolate. * ^3^ CTX-M-1 group gene, except for CTX-M-9 group gene detected in four isolates (three and one isolate of phylogenetic group B2 and D, respectively). * ^4^ CTX-M-1 group gene, except for CTX-M-9 group gene detected in one isolate (phylogenetic group B2).

**Table 2:** Genotypes and antimicrobial resistance profile of representative *E. coli* strains of phylogenetic group A, B1, B2 and D isolated in Cuba (n = 71).

Strain ID (IPK)	Year	Specimen	Patient Age, Sex	Province * ^1^	Phylogenetic Group	ST * ^2^	Allelic Profile * ^3^	O25b Allele * ^4^	*fimH* Type * ^4^	Beta-Lactamase Gene	PMQR * ^5^ Gene	Antimicrobial Resistance Profile * ^6^
25	2014	wound	Adult, F	LH	A	ST1463	6-95-4-222-7-7-7	ND	ND	*bla*_TEM-1_, *bla*_CMY-2_		CAZ, CTX, CXM, FOX, TOB
86	2014	blood	Adult, F	LH	A	ST5715 (ST410 SLV)	6-4-12-1-20-18-73	ND	ND	*bla*_TEM-1_	*qnrB*	CAZ, CTX, CXM, ATM, NAL, CIP, NOR, GEN, TOB, AMK, SXT
110	2015	peritoneal fluid	Adult, M	LH	A	ST167 (ST10 SLV)	10-11-4-8-8-13-2	ND	ND	*bla*_CTX-M-15_	*aac (6’)-Ib-cr, qnrB*	TZP, CTX, CXM, FOX, ATM, NAL, CIP, NOR
118	2015	wound	Adult, M	SC	A	ST1488 (ST10 SLV)	10-11-4-8-8-8-73	ND	ND	*bla*_CTX-M-2_	*qnrS*	CTX, CXM, TOB, AMK
119	2015	respiratory tissue	Adult, M	VC	A	ST1488 (ST10 SLV)	10-11-4-8-8-8-73	ND	ND	*bla*_CTX-M-15_	*aac (6’)-Ib-cr*	CTX, CXM, ATM, NAL, CIP, NOR, GEN
145	2015	urine	Adult, F	HG	A	ST4238 (ST10 SLV)	10-11-4-8-8-9-2	ND	ND	*bla*_CTX-M-15_, *bla*_TEM-1_	*qnrB*	CTX, CXM, FEP, ATM, GEN, SXT
152	2015	wound	Adult, F	CF	A	ST10 (ST10 Cplx)	10-11-4-8-8-8-2	ND	ND	*bla*_CTX-M-15_	*qnrS*	CTX, CXM, ATM
121	2015	wound	Adult, F	PR	A	ST156 (ST156 Cplx)	6-29-32-16-11-8-44	ND	ND	*bla*_CTX-M-15_, *bla*_TEM-1_	*qnrB*	CTX, CXM, FOX, ATM, NAL, CIP, NOR, GEN
107	2015	urine	Adult, F	LH	A	ST166 DLV	52-746-55-53-40-422-43	ND	ND		*qnrD*	CXM
192	2016	respiratory tissue	Adult, M	HG	A	ST1437	10-27-5-8-8-1-2	ND	ND	*bla*_CTX-M-32_	*qnrB*	CAZ, CTX, FEP, NAL, CIP, GEN, ATM
204	2016	placenta	Adult, F	MT	A	ST1421	8-7-1-8-8-8-2	ND	ND	*bla*_CTX-M-32_		CAZ, CTX, FEP, NAL, CIP, GEN, ATM
266	2016	urine	Adult, F	LH	A	ST410	6-4-12-1-20-18-7	ND	ND	*bla*_TEM-1_	*qnrB*	FOX, FEP, CIP, NOR, NAL, SXT
290	2016	urine	Child, F	HG	A	ST735 SLV	92-11-4-8-8-8-295	ND	ND		*qnrB*	CIP, NOR, NAL
75	2018	urine	Child, M	LH	A	ST410 DLV	6-4-281-1-20-12-7	ND	ND	*bla*_CTX-M-15_	*oqxAB*	NAL, CIP, NOR, SXT
543	2018	blood	Adult, M	SC	A	ST410	6-4-12-1-20-18-7	ND	ND	*bla*_CTX-M-15_	*qnrS*	TZP, CAZ, CTX, FEP, FOF, NAL, CIP, MEM, IPM, GEN, AMK, SXT
556	2018	blood	Child, M	LH	A	ST410 DLV	6-4-281-1-20-12-7	ND	ND	*bla*_CTX-M-15_, *bla*_TEM-1_	*aac (6’)-Ib-cr, oqxAB*	TZP, CAZ, CTX, NAL, CIP, GEN, AMK, SXT
17	2014	urine	Adult, F	SS	B1	ST156	6-29-32-16-11-8-44	ND	ND	*bla*_TEM-1_	*qnrB*	CAZ, SXT
101	2015	respiratory tissue	Adult, M	LH	B1	ST641 (ST86 Cplx)	9-6-33-131-24-8-7	ND	ND	*bla*_CTX-M-15_	*aac (6’)-Ib-cr, qnrB*	CTX, CXM, ATM
182	2016	urine	Adult, M	SC	B1	ST448	6-6-5-16-11-8-7	ND	ND	*bla*_NDM-1_, *bla*_CTX-M-15_		CXM, TZP, CAZ, CTX, FOX, FEP, NAL, CIP, NOR, MEM, IPM
184	2016	kidney	Adult, M	VC	B1	ST448	6-6-5-16-11-8-7	ND	ND	*bla*_NDM-1_, *bla*_CTX-M-15_		CXM, CTX, FEP, NAL, CIP, FOF, TOB, ATM, MEM, IPM
191	2016	sputum	Adult, M	HG	B1	ST162 (ST469 Cplx)	9-65-5-1-9-13-6	ND	ND	*bla*_CTX-M-15_		CXM, CTX, FEP, NAL, CIP, FOF, TOB, ATM
216	2016	wound	Child, F	SC	B1	ST23 (ST23 Cplx)	6-4-12-1-20-13-7	ND	ND	*bla*_CTX-M-15_, *bla*_TEM-1_	*aac (6’)-Ib-cr*	CTX, FEP, NAL, CIP, NOR, GEN, SXT, TIC
152	2018	urine	Adult, F	LH	B1	ST224	6-4-33-16-11-8-6	ND	ND			CTX, CIP, NOR, SXT
185	2018	urine	Adult, M	LH	B1	ST448	6-6-5-16-11-8-7	ND	ND	*bla*_CMY-2_	*aac (6’)-Ib-cr*	CTX, CIP, NOR, SXT
232	2018	skin	Adult, F	LH	B1	ST448	6-6-5-16-11-8-7	ND	ND	*bla*_CTX-M-15_	*aac (6’)-Ib-cr*	CAZ, CTX, FEP, CIP, MEM, GEN, SXT
373	2018	skin	Adult, F	LH	B1	ST448	6-6-5-16-11-8-7	ND	ND	*bla*_CTX-M-15_, *bla*_CMY-2_	*aac (6’)-Ib-cr*	TZP, CAZ, CTX, FOX, FEP, CIP, MEM, AMK, SXT
544	2018	blood	Adult, M	SC	B1	ST4173	6-6-32-16-9-7-6	ND	ND	*bla*_CTX-M-15_	*qnrS*	CAZ, CTX, FEP, FOF, CIP, SXT
19	2014	blood	Adult, F	GT	B2	ST5718 (ST131 SLV)	36-40-9-13-17-11-25	-	ND		*qnrD*	SXT
45	2014	endotracheal tube	Adult, M	SC	B2	ST131	53-40-47-13-36-28-29	O25b	*fimH-30*	*bla*_CTX-M-27_	*aac (6’)-Ib-cr, qnrS*	CAZ, CTX, CXM, ATM, CIP, NOR
68	2014	respiratory tissue	Adult, M	LH	B2	ST131	53-40-47-13-36-28-29	O25b	*fimH-30*	*bla*_CTX-M-27_		CAZ, CTX, CXM, FOX, FEP, ATM, NAL, CIP, NOR, MEM, IPM, CST, TOB, SXT
69	2014	respiratory tissue	Adult, M	LH	B2	ST131	53-40-47-13-36-28-29	*-*	ND	*bla*_CTX-M-27_	*aac (6’)-Ib-cr, qnrB*	CAZ, CTX, CXM, FEP, ATM, NAL, CIP, NOR, TOB, SXT
89	2014	tracheal aspirate	Adult, M	LH	B2	ST131	53-40-47-13-36-28-29	*-*	ND	*bla*_CTX-M-15_	*aac (6’)-Ib-cr*	TZP, CAZ, CTX, ATM, NAL, CIP, NOR, MEM, GEN, TOB, SXT
104	2015	wound	Adult, M	LH	B2	ST131	53-40-47-13-36-28-29	*-*	ND	*bla*_CTX-M-15_	*aac (6’)-Ib-cr*	CTX, CMX, FEP, ATM, NAL, CIP, NOR, MEM, GEN, TOB, SXT
117	2015	wound	Adult, F	SC	B2	ST131	53-40-47-13-36-28-29	O25b	*fimH-30*	*bla*_CTX-M-15_, *bla*_TEM-1_	*aac (6’)-Ib-cr*	CTX, CXM, ATM, NAL, CIP, NOR, GEN
130	2015	urine	Adult, F	VC	B2	ST5717 (ST131 SLV)	53-40-47-13-36-28-73	-	ND	*bla*_CTX-M-15_, *bla*_TEM-1_	*aac (6’)-Ib-cr*	CAZ, CTX, CXM, FEP, ATM, NAL, CIP, NOR, SXT
151	2015	catheter tip	Adult, M	CF	B2	ST131	53-40-47-13-36-28-29	O25b	*fimH-30*	*bla*_CTX-M-27_		CTX, CXM, FEP, NAL, CIP, NOR, SXT
168	2015	urine	Adult, F	HG	B2	ST5716 (ST131 SLV)	53-24-47-13-36-28-29	O25b	*fimH-30*	*bla*_CTX-M-15_	*aac (6’)-Ib-cr*	CTX, CXM, FEP, NAL, CIP, NOR, TOB, SXT
177	2016	respiratory tissue	Adult, M	VC	B2	ST131	53-40-47-13-36-28-29	O25b	*fimH-30*	*bla*_CTX-M-27_		CAZ, CXM, CTX, FEP, NAL, CIP
186	2016	urine	Child, M	CF	B2	ST131	53-40-47-13-36-28-29	O25b	*fimH-30*	*bla*_CTX-M-27_		CXM, CTX, FEP, NAL, CIP
203	2016	catheter	Adult, F	MT	B2	ST131	53-40-47-13-36-28-29	O25b	*fimH-30*	*bla*_CTX-M-27_		SXT, CXM, CAZ, CTX, FEP, NAL, CIP
222	2016	urine	Adult, F	LH	B2	ST131	53-40-47-13-36-28-29	O25b	*fimH-30*	*bla*_CTX-M-15_	*aac (6’)-Ib-cr*	CMX, CTX, FOX, FEP, NAL, CIP, NOR, MEM, IPM, TOB, AMK, SXT
234	2016	wound	Adult, M	LH	B2	ST131	53-40-47-13-36-28-29	*-*	ND	*bla*_CTX-M-15_, *bla*_TEM-1_	*aac (6’)-Ib-cr, qnrB*	GEN, CTX, CIP
283	2016	endotracheal tube	Adult, M	LH	B2	ST131	53-40-47-13-36-28-29	O25b	*fimH-30*	*bla*_CTX-M-27_		CAZ, CTX, NAL, CIP, NOR, SXT
288	2016	urine	Child, F	HG	B2	ST3185 (ST131 SLV)	92-40-47-13-36-28-29	O25b	*fimH-30*	*bla*_CTX-M-15_	*qnrS*	CTX, FEP, NAL, CIP
324	2016	blood	Adult, M	LH	B2	ST131	53-40-47-13-36-28-29	-	ND		*aac (6’)-Ib-cr*	CAZ, FEP, NAL, CIP, NOR, MEM, IPM, GEN, SXT, CST
37	2018	urine	Adult, F	LH	B2	ST131	53-40-47-13-36-28-29	O25b	*fimH-30*	*bla*_CTX-M-27_		CAZ, ATM, CIP, NOR, SXT
149	2018	urine	Adult, M	LH	B2	ST131	53-40-47-13-36-28-29	O25b	*fimH-30*	*bla*_CTX-M-27_		CAZ, CIP, NOR, SXT
194	2018	urine	Adult, F	LH	B2	ST3223 (ST131 SLV)	10-40-47-13-36-28-29	O25b	*fimH-30*	*bla*_CTX-M-15_	*aac (6’)-Ib-cr, qnrB*	CAZ, ATM, CIP, NOR
330	2018	urine	Adult, M	LH	B2	ST131	53-40-47-13-36-28-29	O25b	*fimH-30*	*bla*_CTX-M-15_	*Aac (6’)-Ib-cr, qnrB*	ATM, CIP, NOR, SXT
398	2018	wound	Child, F	CF	B2	ST131	53-40-47-13-36-28-29	O25b	*fimH-30*	*bla*_CTX-M-27_		TPZ, CAZ, CTX, FEP, CIP, GEN, AMK, SXT
401	2018	endotracheal tube	Child, M	CF	B2	ST131	53-40-47-13-36-28-29	O25b	*fimH-30*	*bla*_CTX-M-15_		CAZ, CTX, FOX, FEP, CIP, MEM, IPM, GEN, AMK, SXT
417	2018	skin	Adult, F	LH	B2	ST131	53-40-47-13-36-28-29	-	ND	*bla*_CTX-M-27_		CAZ, CTX, FEP, CIP, SXT
506	2018	skin	Adult, F	LH	B2	ST131	53-40-47-13-36-28-29	O25b	*fimH-30*	*bla*_CTX-M-27_		CAZ, CTX, FEP, CIP, SXT
528	2018	sputum	Adult, M	IJ	B2	ST131	53-40-47-13-36-28-29	*-*	ND	*bla*_CTX-M-15_	*aac (6’)-Ib-cr*	CAZ, CTX, FOX, FEP, CIP, MEM, IPM, GEN, AMK, SXT
602	2018	urine	Adult, F	IJ	B2	ST131	53-40-47-13-36-28-29	O25b	*fimH-30*	*bla*_CTX-M-27_, *bla*_TEM-1_		CAZ, NAL, CIP, SXT
610	2018	urine	Adult, F	IJ	B2	ST131	53-40-47-13-36-28-29	*-*	ND	*bla*_CTX-M-27_		CAZ, NAL, CIP, SXT
629	2018	lochia	Adult, F	GT	B2	ST1193	14-14-10-200-17-7-10	*-*	ND	*bla*_CTX-M-27_		CAZ, CTX, FEP, CIP, SXT
15	2014	wound	Child, F	HG	D	ST5162 (ST405 SLV)	35-37-29-25-4-5-2	ND	ND	*bla*_TEM-1_	*qnrB*	CXM, GEN
65	2014	respiratory tissue	Adult, F	LH	D	ST405	35-37-29-25-4-5-73	ND	ND	*bla*_CTX-M-15_		TZP, CAZ, CTX, CXM, FOX, FEP, ATM, NAL, CIP, NOR, GEN, TOB, SXT
174	2015	cerebrospinal fluid	Adult, M	LH	D	ST405	35-37-29-25-4-5-73	ND	ND	*bla*_CTX-M-55_	*aac (6’)-Ib-cr*	TZP, CAZ, CTX, FOX, FEP, FOF, GEN, CIP, SXT
217	2016	urine	Adult, F	LH	D	ST3496 (ST405 DLV)	35-37-29-382-4-8-73	ND	ND	*bla*_CTX-M-15_		SXT, CXM, CTX, FOX, FEP, NAL, CIP, NOR, FOF
258	2016	urine	Child, F	LH	D	ST405	35-37-29-25-4-5-73	ND	ND	*bla*_CTX-M-14_		CFZ, NAL, CIP, SXT
261	2016	cerebrospinal fluid	Adult, F	AT	D	ST349	34-36-39-87-67-16-4	ND	ND		*qnrS*	SXT
274	2016	blood	Adult, M	SC	D	ST648	92-4-87-96-78-58-2	ND	ND	*bla*_CTX-M-15_, *bla*_TEM-1_	*qnrS*	TZP, CAZ, CTX, FOX, FEP, ATM, CIP, NOR, NAL, SXT
135	2017	urine	Adult, F	SC	D	ST405	35-37-29-25-4-5-73	ND	ND	*bla*_CTX-M-15_	*aac (6’)-Ib-cr*	TZP, CTX, FOX, FEP, NAL, CIP, MEM, GEN, SXT
517	2017	lung tissue	Child, M	CF	D	ST405	35-37-29-25-4-5-73	ND	ND	*bla*_CTX-M-15_		CAZ, FOX, FEP, NAL, CIP, MEM, IPM, GEN, TET, SXT
148	2018	urine	Adult, F	LH	D	ST69 (ST69 Cplx)	32-35-27-6-5-5-4	ND	ND	*bla*_TEM-1_		NAL
537	2018	urine	Adult, F	SC	D	ST405	35-37-29-25-4-5-73	ND	ND	*bla*_TEM-1_		NAL, CIP, GEN
605	2018	urine	Adult, F	IJ	D	ST69 (ST69 Cplx)	21-35-27-6-5-5-4	ND	ND	*bla*_TEM-1_		NAL, SXT
622	2018	blood	Adult, F	IJ	D	ST68	33-26-2-31-5-16-19	ND	ND	*bla*_CTX-M-15_, *bla*_TEM-1_		TZP, CTX, GEN, AMK, SXT
630	2018	blood	Adult, F	IJ	D	ST69 (ST69 Cplx)	21-35-27-6-5-5-4	ND	ND	*bla*_TEM-1_		GEN, NAL

* ^1^ Abbreviations of provinces: AT, Artemisa; CF, Cienfuegos; GT, Guantánamo; HG, Holguín; IJ, Isla de la Juventud; LH, La Habana; MT, Matanzas; PR, Pinar del Río; SC, Santiago de Cuba; SS, Sancti Spíritus; VC, Villa Clara. * ^2^ SLV, single-locus variant; DLV, double-locus variant; Cplx, complex. * ^3^ Underline represents variant locus number of SLV or DLV shown in the left column. * ^4^ ND, not determined; −, negative. * ^5^ Plasmid-mediated quinolone resistance. * ^6^ Abbreviations of antimicrobials: AMK, amikacin; ATM, aztreonam; CAZ, ceftazidime; CIP, ciprofloxacin; CST, colistin; CTX, cefotaxime; CXM, cefuroxime; FEP, cefepime; FOF, fosfomycin; FOX, cefoxitin; GEN, gentamicin; IPM, imipenem; MEM, meropenem; NAL, nalidixic acid; NOR, norfloxacin; SXT, trimethoprim-sulfamethoxazole; TOB, tobramycin; TZP, piperacillin-tazobactam.

## Supplementary Materials

The following are available online at https://www.mdpi.com/2076-0817/9/1/65/s1, Table S1: Coexistence of beta-lactamase genes and PMQR genes in *E. coli* isolates, Table S2: Mutations in QRDR of GyrA and ParC in ExPEC isolates showing quinolone resistance, Table S3: Primers used for sequencing in this study.
