# Supplementary material for: High Prevalence of CTX-M Type Extended-Spectrum Beta-Lactamase Genes and Detection of NDM-1 Carbapenemase Gene in Extraintestinal Pathogenic Escherichia coli in Cuba
Source: Pathogens. 2020 Jan 16;9(1):65. doi: 10.3390/pathogens9010065 (PMC7168674; doi:10.3390/pathogens9010065)
Supplement: Supplementary file 1 [file pathogens-09-00065-s001.pdf]

pathogens

MDPI

Table S1. Coexistence of beta-lactamase genes and PMQR genes in *E. coli* isolates.

Beta-Lactamase Gene, PMQR Determinant	Phylogenetic Group				Total	Prevalence (%) Among	
	A (n = 64)	B1 (n = 21)	B2 (n = 150)	D (n = 71)	(n = 306)	CTX-M-positive isolates	TEM-positive isolates
CTX-M	39	11	94	43	187		
TEM	16	7	47	27	97		
CTX-M + *aac (6')-Ib-cr*	22	7	57	16	102	54.5	
CTX-M + *qnrB*	2	1	0	2	5	2.7	
CTX-M + *qnrS*	1	1	3	1	6	3.2	
TEM + *qnrB*	2	1	0	2	5		5.2
TEM + *qnrS*	1	0	0	1	2		2.1

pathogens

MDPI

Table S2. Mutations in QRDR of GyrA and ParC in ExPEC isolates showing quinolone resistance.

Strain ID (IPK-No.)	Province	Phylogenetic Group	O25b	PMQR Gene	Mutation in QRDR	
					GyrA	ParC
17	Santi Spiritus	B1		*qnrB*	S83L, D87N	-
27	Santi Spiritus	B2	O25b	-	S83L, D87N	S80I, E84V
46	Santiago de Cuba	B2	O25b	-	S83L, D87N	S80I, E84V
52	Guantánamo	B2	O25b	-	-	S80R
60	La Habana	D		-	S83L, D87N	S80I, E84G
64	La Habana	B2	O25b	*aac (6′)-Ib-cr*	S83L, D87N	S80I, E84V
67	La Habana	D		-	S83L, D87N	S80I, E84G
71	La Habana	B2	O25b	*aac (6′)-Ib-cr*	S83L	S80I, E84V
77	La Habana	A		*aac (6′)-Ib-cr*	D87N	S80I
79	La Habana	D		-	S83L	-
80	La Habana	B1		-	-	S80I, E84V
86	La Habana	A		*qnrB*	S83L, D87N	S80I
89	La Habana	B2		*aac (6′)-Ib-cr*	S83L, D87N	S80I, E84V
90	La Habana	D		*aac (6′)-Ib-cr*	S83L, D87N	S80I
91	La Habana	D		-	S83L, D87N	S80I
98	La Habana	B2	O25b	*aac (6′)-Ib-cr*	S83L, D87N	S80I, E84V
103	La Habana	B1		-	S83L, D87N	S80I
104	La Habana	B2		*aac (6′)-Ib-cr*	S83L, D87N	S80I, E84V
106	La Habana	B2		*aac (6′)-Ib-cr*	S83L, D87N	S80I, E84V
108	La Habana	B2	O25b	-	S83L, D87N	S80I, E84V
109	La Habana	B2	O25b	-	S83L, D87N	S80I, E84V
117	Santiago de Cuba	B2		*aac (6′)-Ib-cr*	S83L, D87N	S80I, E84V
119	Villa Clara	A		*aac (6′)-Ib-cr*	S83L, D87N	S80I
120	Pinar del Río	A		-	S83L, D87N	S80I
122	Villa Clara	B2	O25b	*aac (6′)-Ib-cr*	S83L, D87N	S80I, E84V
130	Villa Clara	B2		*aac (6′)-Ib-cr*	S83L, D87N	S80I, E84V
132	Villa Clara	B2	O25b	*aac (6′)-Ib-cr*	S83L, D87N	S80I, E84V
133	Villa Clara	D		*aac (6′)-Ib-cr, qnrB*	S83L, D87N	S80I, E84V
146	Holguín	B2	O25b	-	D87Y	-
150	Santiago de Cuba	B2	O25b	*aac (6′)-Ib-cr*	S83L, D87N	S80I, E84V
151	Cienfuegos	B2	O25b	-	S83L, D87N	S80I, E84V
154	Cienfuegos	D		*aac (6′)-Ib-cr*	S83L, D87N	S80I
155	Cienfuegos	B2		-	D87S	S80I
163	Villa Clara	B2	O25b	-	S83L, D87N	S80I, D84S
168	Holguín	B2	O25b	*aac (6′)-Ib-cr*	S83L, D87N	S80I, E84V
171		B2	O25b	*aac (6′)-Ib-cr*	S83L, D87N	S80F, E84V
173	Villa Clara	B2	O25b	*aac (6′)-Ib-cr*	S83L, D87N	S80I, E84V
174	La Habana	D		*aac (6′)-Ib-cr, qnrB*	S83L, D87N	S80I

−, negative. The most common types of mutations were shaded.

pathogens

MDPI

Table S3. Primers used for sequencing in this study.

Target Gene (*bla*)	Primer	Sequence (5'-3')	Product Size
TEM	TEM-F1	ATATTGAAAAGGAAGAGTATG	1 kb (+TEM-R1)
	TEM-R1	AGTAAACTTGGTCTGACAGT	
	TEM-F2	ATGAGTATTCAACATTTTCG	
	TEM-R2	TACCAATGCTTAATCAGTGA	
	TEM-R3	GTGACTGGTGAGTACTCAAC	
	TEM-F3	CACAACATGGGGGATCATGT	
CTX-M-1-Group	CTX-M-15p	GGTTAAAAAATCACTGCG	1 kb (+CTX-M-15R1)
	CTX-M-15-R1	TTATGGCCTGGTATGCGCAAGC	
	CTX-M-15-R2	GCAAAGCGCTCATCAGCACG	
CTX-M-9-Group	CTX-M-27-F1	TTACAATGTGTGATAAGCAGTC	1.1 kb (+CTX-M-27-R1)
	CTX-M-27-R2	TTGAACTTTTGCTTTGCCACGG	
	CTX-M-27-R1	ACGTCTCATCGCCGATCGCG	
	CTX-M-27-F2	ATGACGCTGGCAGAACTGAG	
CMY	CMY-F1	ATGATGAAAAAATCGTTATGC	1150 bp (+CMY-R1)
	CMY-R1	GTCAGTTATTGCAGCTTTTCAAG	
	CMY-R2	TGCTGCGTGACTGGGTGGTT	
	CMY-F2	CCGTACACGTTTCTCCGGGACA	
NDM	NDM-Aba	TTGCTCAGCTTGTTGATTATCATATGGC	1.2kb (+NDM-BleR2)
	NDM-BleR2	ATCGAGATCATCCAACCGCA	
	NDM-F1	GGTGGCTGCCTGATCAAGGA	
	NDM-R1	CGATCAAACCGTTGGAAGCGACT	
